# Existential suffering at the end of life in long-term care: A critical essay on recognition, dignity, and witness

**DOI:** 10.1177/26323524261469210

**Published:** 2026-07-13

**Authors:** Anat Romem, Rachel Bardach

**Affiliations:** 1 Henrietta Szold School of Nursing, Faculty of Medicine, Hadassah & the Hebrew University, Jerusalem, Israel; 2 26733 Herzog Medical Center, Jerusalem, Israel

**Keywords:** existential suffering, long-term care, palliative care, dignity, existential loneliness, spiritual care, older adults, witness

## Abstract

Existential suffering is widely acknowledged in palliative care, but it remains inconsistently recognized and unevenly addressed in long-term care. This critical essay argues that the gap is not caused by clinician indifference, but by a persistent mismatch between whole-person ideals and care systems organized around measurable symptoms, risk management, task completion, and professional uncertainty. Drawing on literature on suffering, existential loneliness, dignity, spiritual care, and recent critiques of impersonal care systems, the essay identifies three interrelated shortcomings: the reduction of suffering to medically actionable symptoms; the depersonalizing effects of institutional routines on frail older adults; and the absence of shared existential literacy across interdisciplinary teams. The essay also cautions against romanticizing suffering or treating meaning-making as a clinical expectation. It proposes a practical framework of recognition, dignity, and witness: recognizing biography and identity as clinically relevant; protecting dignity in the ordinary details of care; and enacting witness through disciplined presence, documentation, referral, and team accountability. Long-term care cannot resolve every existential wound, but it can reduce existential neglect by treating personhood as a core quality indicator of palliative care.

## Plain language summary

Palliative care is meant to support the whole person, not only treat disease or physical symptoms. However, people who are nearing the end of life may experience suffering that is not only physical. They may feel lonely, dependent, ashamed, afraid, forgotten, or no longer recognized as the person they once were. This kind of suffering is often called existential suffering. This essay focuses on older adults living in long-term care settings, where many people spend the last stage of life. In these settings, care is often organized around safety, medication, hygiene, nutrition, monitoring, and symptom control. These are all important. However, they may not be enough. A person can receive good physical care and still feel unseen, powerless, or deeply alone. The essay argues that existential suffering should not be treated as a vague, secondary, or optional concern. It should be recognized as a real part of end-of-life care. The essay identifies several problems. First, healthcare systems often respond more easily to suffering that can be measured, such as pain or breathing difficulty. Second, staff may not always have enough training or confidence to recognize and respond to emotional, spiritual, and existential distress. Third, responsibility for this type of suffering may be unclear, with nurses, doctors, social workers, psychologists, and spiritual care providers all involved but not always working from a shared approach. The essay suggests that long-term care teams need better “existential literacy.” This means the ability to notice, name, discuss, document, and respond to signs of existential suffering. Care should include attention to the person’s life story, values, relationships, dignity, and need for human presence.

## Introduction: The argument of this critical essay

Palliative care has long defined itself through whole-person care, yet existential suffering remains one of the places where that promise is least consistently translated into routine practice. The problem is not that clinicians do not care. It is that long-term care systems are usually organized around what can be measured, documented, prioritized, and acted upon quickly. Pain, dyspnea, delirium, nausea, falls, pressure injuries, and medication safety generate clear clinical responses. Statements such as “I am no longer myself,” “I am waiting for nothing,” “I feel forgotten,” or “this is not living” are often heard with sympathy, but they do not always produce the same level of structured clinical attention.

This essay argues that existential suffering at the end of life in long-term care should be treated as a core clinical, relational, and moral concern, not as an optional spiritual add-on. Cassell’s classic definition of suffering as distress that threatens the integrity of the person remains central because it makes clear that the object of care is not only the diseased body but the person whose continuity, identity, role, and future are threatened.^1^ O’Connor similarly notes that end-of-life suffering extends beyond physical symptoms and is often difficult for clinicians to name and address.^2^ The recent Lancet Oncology Commission on the “human crisis” in cancer, although focused on oncology, makes a parallel argument: technically sophisticated care can still fail when systems become fragmented, impersonal, and insufficiently relational.^3^ The same cultural failure is visible in long-term care, where many older adults die slowly, dependently, and institutionally.

## Symptom control becomes the dominant language of suffering

Physical symptom relief is not the enemy of existential care. In many cases, it is the first ethical obligation. Severe pain, breathlessness, or delirium can intensify fear, isolation, loss of control, and spiritual distress. The real problem is narrower: symptom control often becomes the sole organizing principle of care, while suffering that is relational, biographical, spiritual, or identity-based remains less visible.

This hierarchy of clinical legitimacy is partly conceptual. Existential suffering is difficult to define, and related terms such as existential distress, demoralization, spiritual distress, hopelessness, loneliness, and depression may overlap without being identical.^4,5^ Empirical work with palliative care providers shows that clinicians perceive existential distress as common and serious, but also as difficult to assess and variably treated, often requiring interdisciplinary coordination rather than a single protocol.^6^ Studies in advanced illness further show that existential distress may coexist with depression and anxiety but can also arise from questions of meaning, autonomy, dignity, and relational rupture.^7^

The consequence is practical. A resident who reports pain receives a pain score, medication review, and follow-up. A resident who says, “I am a burden,” may receive reassurance but not necessarily assessment, documentation, team discussion, or a care plan. This difference does not reflect a lack of compassion; it reflects a clinical culture in which measurable suffering is easier to legitimize than suffering that threatens identity and meaning. A palliative culture that claims whole-person care must close this gap.

## Institutional routines can erode personhood

Long-term care settings are shaped by necessary priorities: safety, hygiene, nutrition, medication administration, fall prevention, staffing routines, and regulatory accountability. These priorities protect vulnerable people. Yet they may also create an organizational culture in which residents gradually become known more through their dependency than through their biography. The person who once led a family, practiced a profession, taught, prayed, created, decided, or cared for others may become primarily described through continence status, transfer ability, swallowing risk, agitation, skin integrity, and medication burden.

This is where functional decline becomes biographical and moral. “Moral” here does not mean moral judgment; it means value-laden. Functional losses matter because they touch what the person values: privacy, authority, reciprocity, modesty, memory, spirituality, family role, and self-recognition. In long-term care, bodily dependency can become humiliating when intimate care is rushed, privacy is interrupted, choices are minimized, or former identity disappears from ordinary conversation.

Research on existential loneliness among older adults supports this point. Larsson and colleagues’ qualitative work across residential care, home care, and specialized palliative care found that existential loneliness is not merely social isolation; it is bound to experiences of being unseen, disconnected, dependent, and unable to share one’s deepest concerns.^8^ Edberg and colleagues similarly showed that healthcare professionals recognize existential loneliness among older people but often struggle to know how to respond within everyday care structures.^9^ These studies make the cultural issue concrete: existential suffering is not located only inside the patient. It is shaped by environments that either recognize or obscure personhood.

Therefore, waiting, institutional routines, and dependency should be understood at two levels. Organizationally, they are conditions of care delivery. Existentially, they become sources of distress when they communicate that the person’s time, privacy, voice, or history no longer matters. The same task can either preserve or erode dignity depending on how it is done.

## Existential literacy is not yet a routine team competency

The third shortcoming is professional and organizational: many teams lack a shared language for existential suffering. Nurses, physicians, nursing assistants, social workers, psychologists, chaplains, spiritual care providers, and family caregivers may all encounter it, but responsibility is often diffuse. When everyone is generally responsible, no one is reliably accountable.

Tarbi and colleagues argue that existential care belongs in daily nursing practice rather than only in specialist encounters.^10^ This is especially important in long-term care, where nursing and care aide presence is continuous and where distress often appears in small moments: refusal of care, silence, anger, shame during bathing, repeated questions about death, loss of appetite, or statements of worthlessness. Specialist spiritual care remains essential, but referral alone cannot carry the full burden. Existential suffering often appears before referral, between visits, and during routine care.

Existential literacy means the ability to notice, name, tolerate, document, and respond to existential distress without immediately reducing it to depression, non-compliance, or “normal aging.” It also means knowing when additional assessment is needed. Depression, delirium, pain, medication effects, anxiety, social abandonment, spiritual struggle, and existential distress can interact. The ethical task is not to choose one explanation too quickly, but to hold several possibilities long enough to respond accurately.

A team with existential literacy would ask different questions: What loss is being expressed? What identity is threatened? Is the person ashamed, afraid, lonely, spiritually unsettled, or grieving? Is there a family rupture? Does the person want prayer, silence, life review, reconciliation, privacy, or simply someone who can remain present? These questions do not require every clinician to become a psychotherapist. They require existential attentiveness as a routine part of good palliative care.

## A caution: Do not romanticize suffering

A serious response to existential suffering must also avoid romanticizing it. Palliative care sometimes uses the language of meaning, growth, acceptance, and transcendence in ways that can unintentionally burden patients. Some people do find meaning in dying, illness, faith, reconciliation, or legacy. Others experience only fear, exhaustion, humiliation, anger, or emptiness. Varelius warns that suffering should not be treated as inherently meaningful or morally improving.^11^ This warning is crucial in long-term care, where repeated losses may feel less like a reflective existential passage and more like erosion.

The goal is therefore not to demand meaning-making. It is to make room for the patient’s truth. A resident should not have to transform suffering into wisdom for that suffering to be taken seriously. Ethical care permits crying, protest, silence, unfinished relationships, spiritual doubt, and absence of meaning. The clinician’s role is not to impose a redemptive interpretation but to protect the person from being abandoned within that suffering.

## A way forward: Recognition, dignity, and witness

The required shift is from abstract whole-person language to operational practice. We propose three linked practices: recognition, dignity, and witness.

Recognition means treating biography as clinically relevant. Admission assessment and care planning should include identity-bearing information: what matters to the person, what roles shaped their life, what forms of address they prefer, what routines preserve selfhood, what spiritual or cultural practices matter, and what losses feel most threatening. Recognition also means listening for existential markers: burden, shame, hopelessness, not belonging, waiting to die, fear of being forgotten, and loss of self. These should be documented and discussed, not left as informal impressions.

Dignity means protecting personhood in ordinary care. Chochinov’s dignity work remains important because it asks what sustains the person when cure is no longer possible.^12^ In long-term care, dignity is enacted through modesty during bathing, asking permission before touch, explaining procedures, protecting choice in small decisions, avoiding infantilizing language, honoring prayer or silence, enabling meaningful contact, and preserving former identity in how staff speak about and to the resident. These actions should not be viewed as superficial expressions of sentiment, but as meaningful components of care. Rather, they represent clinical behaviors that may reduce preventable existential injury.

Witness is the most abstract concept and therefore requires practical definition. Witnessing is disciplined presence before suffering that may not be fixable. In practice, it can include sitting without rushing after a resident says they are afraid; reflecting the statement rather than immediately reassuring; asking one open question; documenting the concern; bringing it to the interdisciplinary meeting; involving family, spiritual care, psychology, or medical review when appropriate; and returning later so the encounter is not a one-time emotional event. Witnessing is not doing nothing. It is refusing to disappear behind tasks when the patient reveals suffering that cannot be solved quickly.

These practices can be operationalized without creating an unrealistic burden for already stretched teams. First, admission and review templates can include one brief existential prompt, such as “What matters most now?” or “What feels hardest about this stage of life?” Second, staff handovers can include one sentence on personhood, not only risk. Third, interdisciplinary meetings can distinguish symptoms, mood, relationships, spiritual concerns, and environmental triggers instead of collapsing all distress into a general psychosocial category. Fourth, organizations can support staff through reflective spaces, because existential care exposes clinicians to grief, helplessness, and moral distress. Fifth, quality improvement in long-term care should include dignity-sensitive indicators: whether the resident’s preferred name is used, whether privacy is protected, whether meaningful relationships are supported, whether spiritual or cultural practices are enabled, and whether existential concerns are followed up rather than merely overheard. None of these steps solves existential suffering. Together, they make it less likely that suffering will be hidden in plain sight.

## Final considerations

Long-term care will never remove all existential suffering at the end of life. Nor should palliative care pretend that every loss can be repaired by better communication, spiritual care, or dignity therapy. Some suffering is inseparable from dying, dependency, and human boundedness. But existential neglect is not inevitable. It is produced when systems recognize symptoms more readily than persons, tasks more readily than biographies, and safety more readily than meaning.

The challenge is therefore practical as well as embedded in organizational structures and culture. Palliative care must keep symptom relief at its center while refusing to let symptom control become the whole definition of care. It must train teams to recognize existential distress, build documentation and referral pathways, and evaluate whether daily care protects dignity. Above all, it must hold long-term care to the standard it already claims: the resident is not only a body to be maintained, but a person to be recognized, protected, and accompanied until death.

## Data Availability

This critical essay does not report original data.*
